# Publisher Correction: Small GTPases and BAR domain proteins regulate branched actin polymerisation for clathrin and dynamin-independent endocytosis

**DOI:** 10.1038/s41467-021-25458-x

**Published:** 2021-08-23

**Authors:** Mugdha Sathe, Gayatri Muthukrishnan, James Rae, Andrea Disanza, Mukund Thattai, Giorgio Scita, Robert G. Parton, Satyajit Mayor

**Affiliations:** 1 National Centre for Biological Science (TIFR), Bellary Road, Bangalore, 560065 India; 2 Institute for Molecular Bioscience, University of Queensland, Brisbane, QLD 4072 Australia; 3 Centre for Microscopy and Microanalysis, University of Queensland, Brisbane, QLD 4072 Australia; 4 IFOM, Fondazione Istituto FIRC di Oncologia Molecolare, Milan, 20139 Italy; 5 Department of Oncology and Hemato-Oncology, University of Milan, Milan, 20122 Italy; 6 Simons Centre for the Study of Living Machines, National Centre for Biological Sciences (TIFR), Bellary Road, Bangalore, 560065 India; 7 Institute for Stem Cell Biology and Regenerative Medicine, Bellary Road, Bangalore, 560065 India

Correction to: *Nature Communications* 10.1038/s41467-018-03955-w; published online 9 May 2018.

The original version of this Article contains an error in Fig. 1e, in which the representative image panel was inadvertently duplicated from Fig. 1d by the publisher.

The correct representative image version of Fig. 1e is: 
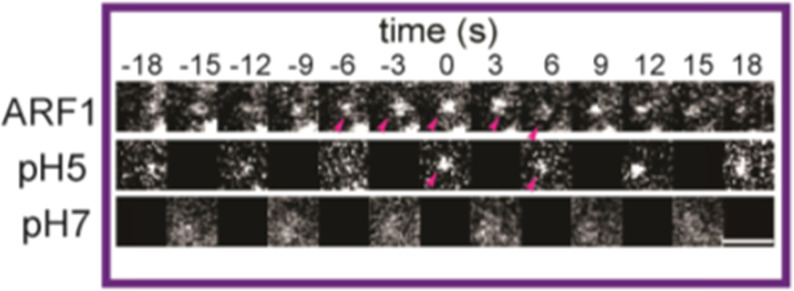


The incorrect version is: 
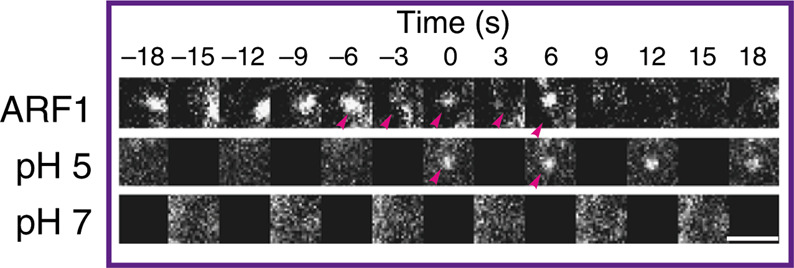


In addition, in Fig. 5b, the dataset used is the same as for Fig. 1d along with its representative image. Both panels 1d and 5b present quantifications of the same CDC42 dataset but from different zones as indicated, to compare with the analysis of IRSp53 dataset in 5a.

Clarifications have been added to the legend of Fig. 5b below to reflect that the same dataset was used in the two quantifications and the legend should read:

“**b** Graphs show the average normalised fluorescence intensity vs. time traces for the recruitment of TagRFPt-CDC42 (dataset taken from Fig. 1d, quantifications done using different zones) to the forming SecGFP-GPI endocytic sites and its corresponding random intensity trace to two different regions [circle, black, *r* = 250 nm; and annulus, orange (*r* = 250–420 nm)]. Error bars, (**a**–**b**) represent s.e.m. (*n*, Table 1). The random traces were derived from randomly assigned spots of the same radius as the endocytic regions, as detailed in S.I. Endocytic distribution at each time point was compared to the random distribution by Mann–Whitney *U* test and the log_10_ (*p*) is plotted below each trace here (**a**) or in Fig. 1d (**b**) [log_10_ (0.05) is −1.3 and log_10_ (0.001) is −2.5]. Representative montages (**a**–**b**) are depicted below the graphs (Note: 5b is reproduced from 1d).”

This original text is:

“**b** Graphs show the average normalised fluorescence intensity vs. time traces for the recruitment of TagRFPt-CDC42 to the forming SecGFP-GPI endocytic sites and its corresponding random intensity trace to two different regions [circle, black, *r* = 250 nm; and annulus, orange (*r* = 250–420 nm)]. Error bars, (**a**–**b**) represent s.e.m. (*n*, Table 1). The random traces were derived from randomly assigned spots of the same radius as the endocytic regions, as detailed in S.I. Endocytic distribution at each time point was compared to the random distribution by Mann–Whitney *U* test and the log_10_ (*p*) is plotted below each trace [log_10_ (0.05) is −1.3 and log_10_ (0.001) is −2.5]. Representative montages are depicted below the graphs.”

