# Supplementary material for: Small GTPases and BAR domain proteins regulate branched actin polymerisation for clathrin and dynamin-independent endocytosis
Source: Nat Commun. 2018 May 9;9:1835. doi: 10.1038/s41467-018-03955-w (PMC5943408; doi:10.1038/s41467-018-03955-w)
Supplement: Supplementary file 1 — Supplementary Information [file 41467_2018_3955_MOESM1_ESM.docx]

**Small GTPases and BAR domain proteins regulate branched actin polymerization for clathrin and dynamin-independent endocytosis**

**Sathe and Muthukrishnan et al**

**Supplementary Figures**

**
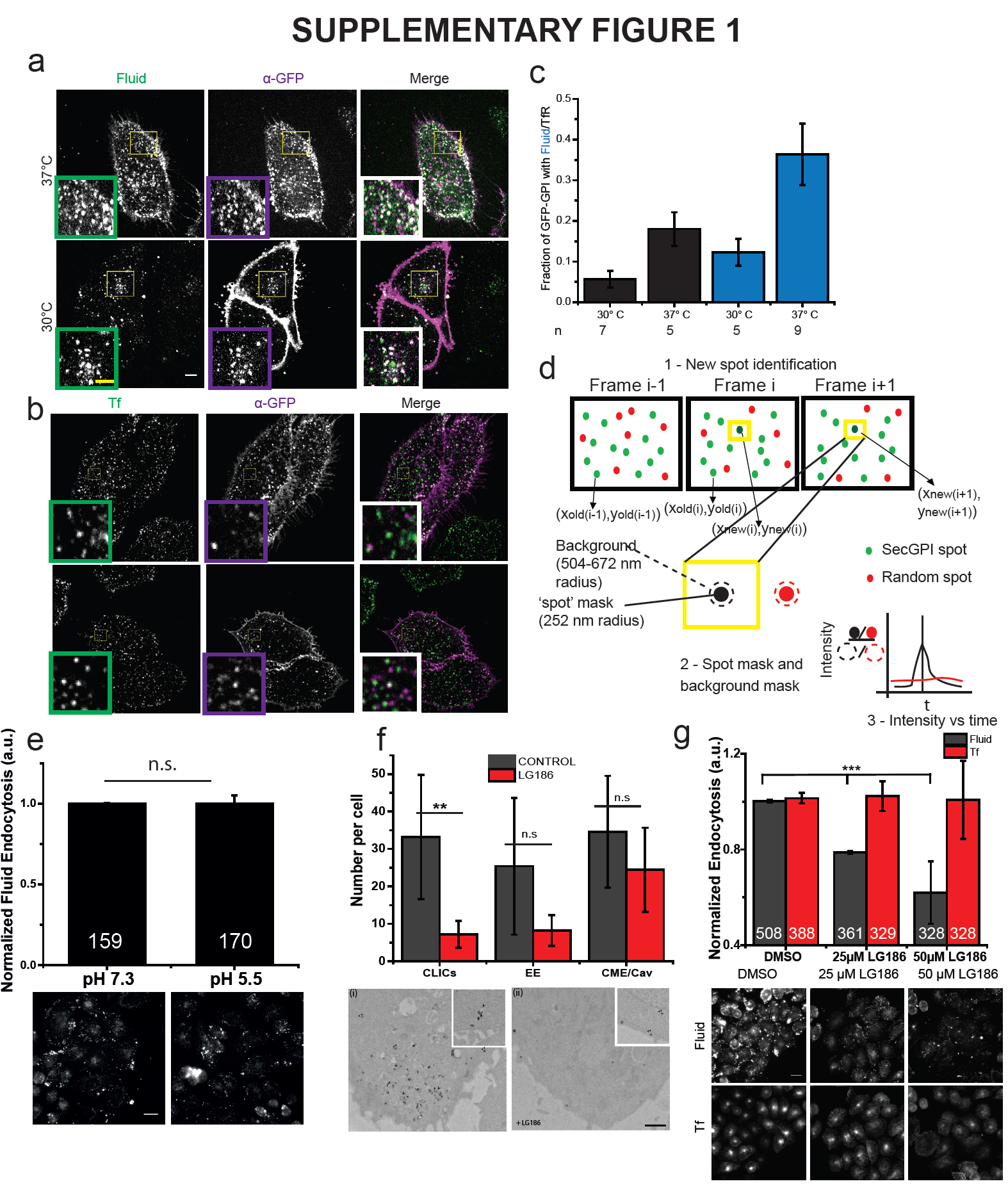
**

**Supplementary Figure 1:** **Characterization of endocytosis in AGS cells.** (**a-b**) Endocytic route of SecGFP-GPI in AGS cells. Single confocal plane shows a 3 minute pulse of α-GFP Fab [**a-b**: magenta in Merge] at 37°C (upper panel) and 30°C (lower panel) along with TMR-dextran (**a**: green in Merge) or A647-Tf (**b**: green in Merge) in AGS cells transiently transfected with SecGFP-GPI. Insets show a magnified view of the marked areas. (**c**) Plot (top) showing quantification of the fraction of GFP-GPI endocytic vesicles containing fluid or Tf. The number of cells is shown below the graph. (**d**) Schematic of pH pulsing analysis, steps (1-3) used for identifying and quantifying the fluorescence spots associated with newly formed endocytic vesicles in the sequential frames of the pH 5 montage. **Step 1** - New spot identification: Each spot (green) in i^th^ frame is compared with the previous frame and is considered new if no nearest neighbour is found by euclidean distance search within 5 pixels (1 pixel = 84nm). Random (red) are generated randomly within the cell mask. **Step 2** - Spot mask (green filled) and background mask (green dashed) considered around the centroid the new spot identified in the step - 1 [also see pH 5 frame in the middle panel of (**Figure 1a**)]. The process is repeated for Random spot mask (red filled) and background mask (red dashed). **Step 3** - schematic of the temporal profile of a new spot (black) and a random spot. Multiple traces pooled from different spots and cells over different days was averaged and shown in rest of the figures. The x-axis represents time and y-axis represents fold change in intensity in the spot over the background. See S.I. for a detailed description. (**e**) AGS cells were pre-treated with either pH 7.3 or pH 5.5 buffer for 5 minutes followed by 5-minute fluid uptake in pH 7.3 buffer. Data was pooled from 2 independent experiments and the number of cells indicated in the graph. (**f**) Untreated AGS (Control, (i)) or LG186-treated AGS (ii) were incubated for 2 minutes at 37°C with 10mg/ml HRP as a fluid phase marker before processing for electron microscopy. Endocytic structures close to the plasma membrane (PM) are filled with the electron dense peroxidase precipitate. Control cells show a range of endocytic structures including vesicular structures (CCP/Cav & EE) (a pair of small arrowheads) and tubular/ring-shaped putative CLIC/GEECs (large arrowhead) but the drug-treated cells show predominant labelling of vesicular profiles. Histogram shows mean endocytic structures quantified per cell (n = 5). CCP/Cav represent vesicles derived from clathrin mediate or caveolar endocytosis and EE represent early endosomes (See S.I.). (**g**) The histogram shows quantification of 5-minute fluid-uptake in AGS cells when treated with indicated concentrations of LG186 or DMSO. Data was pooled from 2 independent experiments and the number of cells indicated in the graph. Scale bar, 5µm (**a-b**), 4µm (**a-b, inset**), 1 µm (**f**) & 20µm (**e and g**) respectively. Error bars (**c**) represent s.e.m. and (**e-g**) s.d. respectively. *p-value* < 0.001 (**) 2-sample student’s T-test (**f**) and Mann-Whitney U test (**e**).


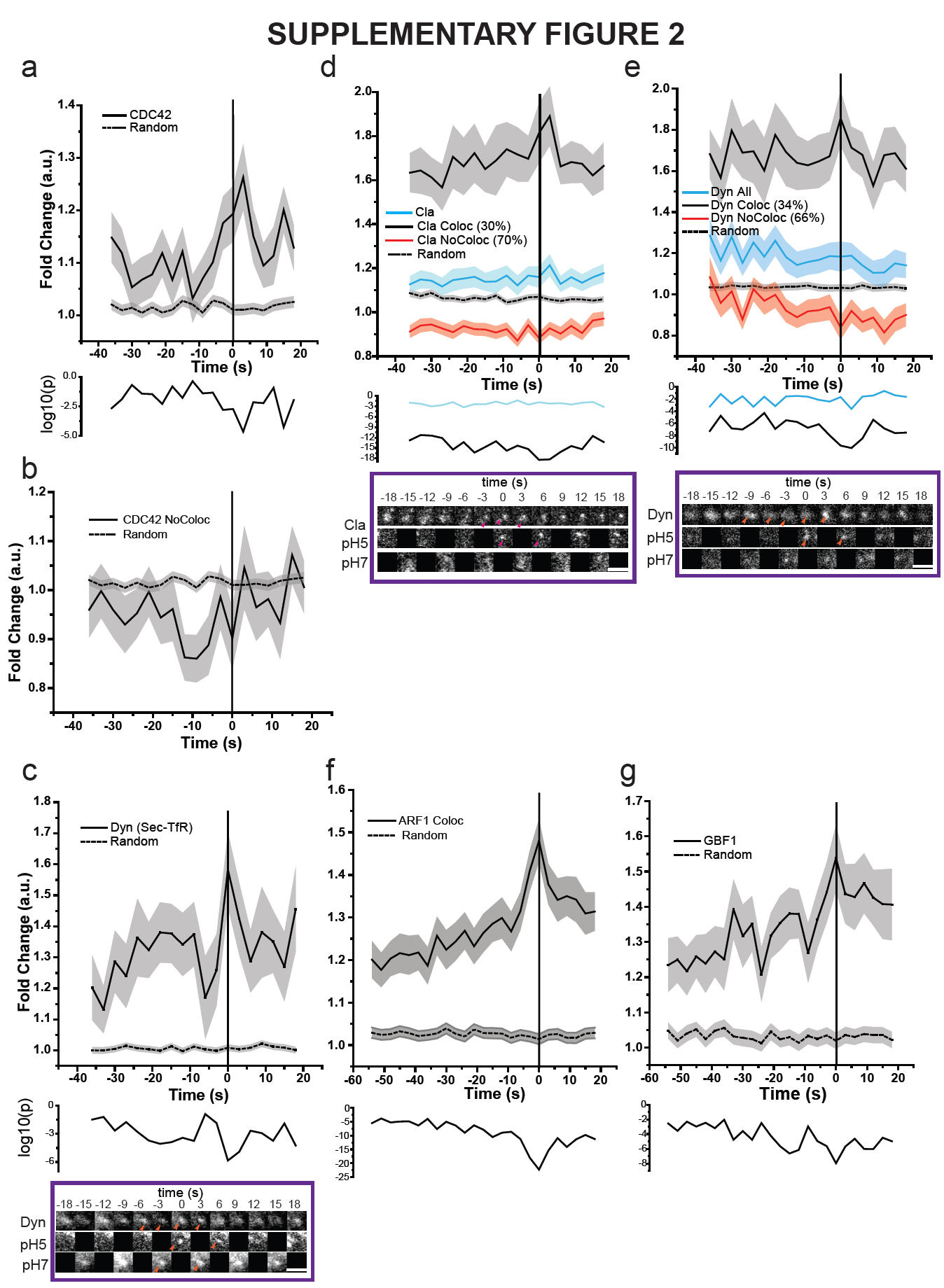


**Supplementary Figure 2:** **Characterization of the pH pulsing assay for visualizing SecGFP-GPI endocytic vesicle formation.** (**a-b**) Graphs show the average normalized fluorescence intensity versus time traces for the recruitment of TagRFPt-CDC42 (CDC42 All) to all forming SecGFP-GPI endocytic sites (**a**) or endocytic sites that do not co-detect TagRFPt-CDC42 (CDC42 NoColoc; 44%) and its corresponding random intensity trace (n, Table 1). (**c-g**) Graphs show the average normalized fluorescence intensity versus time trace for the recruitment of mCherry-dynamin to forming SecTfR endocytic sites [**c**; (n = 21 SecTfR and 1448 random spots from 6 cells, 2 experiments), mCherry-clathrin to forming SecGFP-GPI endocytic sites [**d**; n, Table 1], mCherry-dynamin [**e**; n, Table 1] to forming SecGFP-GPI endocytic sites, mCherry-ARF1 to forming SecGFP-GPI endocytic sites [**f**; (n = 228, as in **Figure 1e**); Note the extended time axis], mCherryGBF1 to forming SecGFP-GPI endocytic sites [**g**; (n = 75, as in **Figure 1e**); Note the extended time axis], and their corresponding random intensity trace. A representative montage depicted below (**c-e**). Arrowheads indicate the spot. The random traces were derived from randomly assigned spots of the same radius as the endocytic regions, as detailed in S.I. Endocytic distribution at each time point was compared to the random distribution by Mann-Whitney U test and the log_10_ (p) [log_10_ (0.05) is -1.3 and log_10_ (0.001) is -2.5] is plotted below each trace (**a**, & **c-g)**. Error bars represent s.e.m. for (**a, & c-g**). Scale bar, 1.5µm (**c-e**).


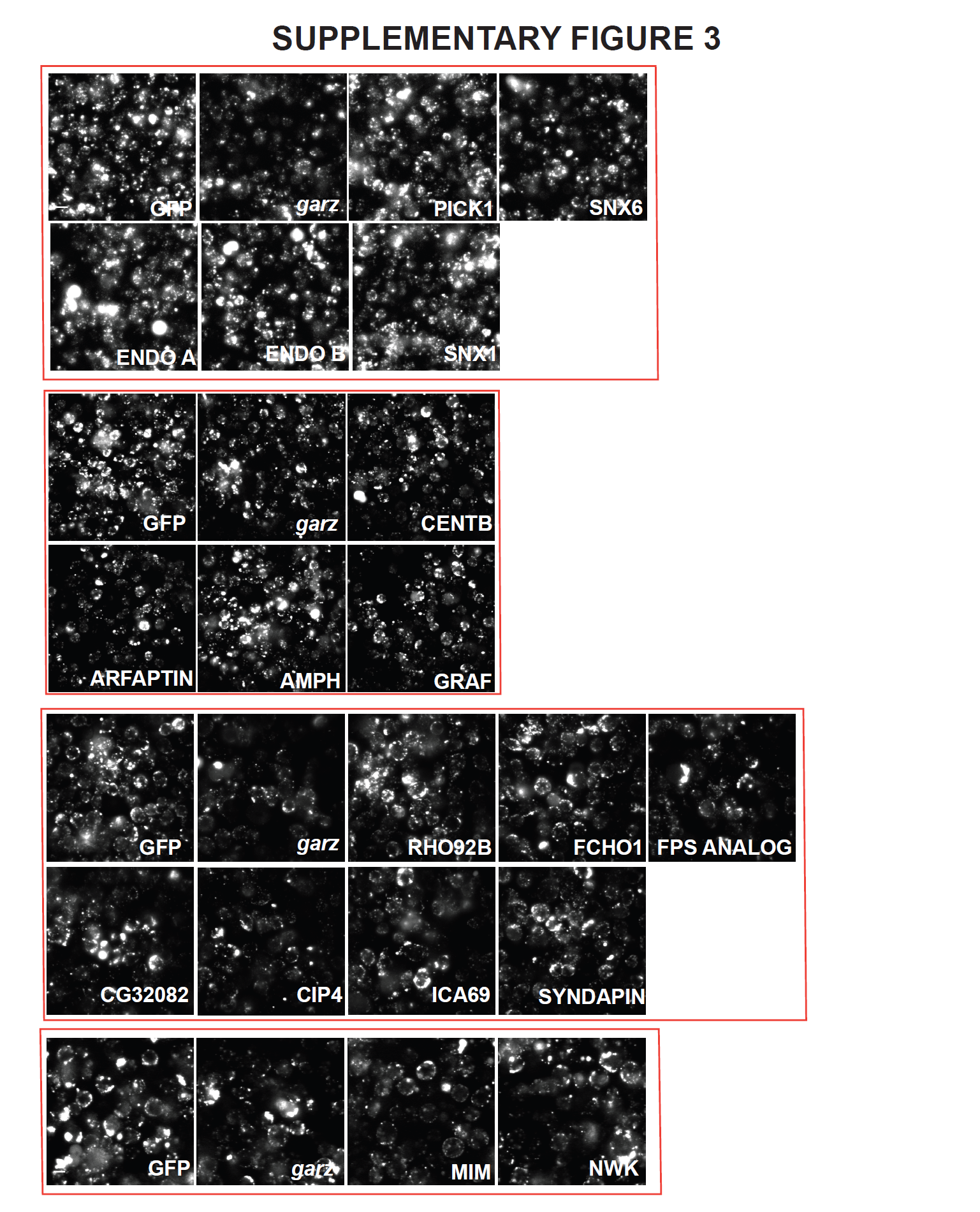


**Supplementary Figure 3: RNAi screen reveals BAR domain proteins involved in CG endocytosis.** Representative images for data shown in Figure 2b. Scale bar is 20µm.


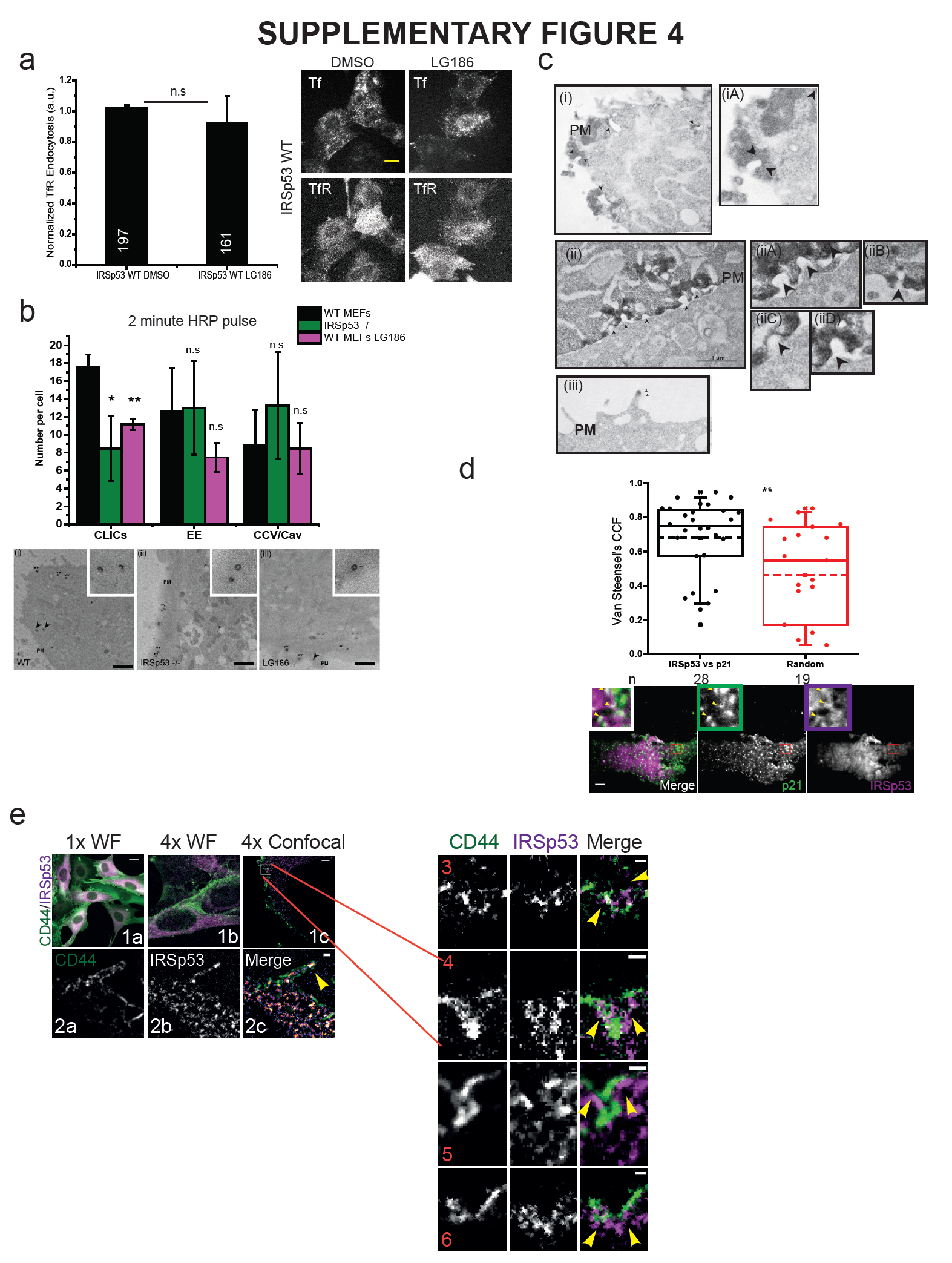
**Supplementary** **Figure 4:** **IRSp53 is involved in CG endocytosis.** (**a**) Histogram (top) showing quantification of mean 5-minute normalized TfR uptake in IRSp53 WT cells when treated with 10µM LG186 for 45 minutes along with the representative images (bottom). Data was pooled from 2 independent experiments and the number of cells is indicated in the figure. (**b**) Histogram (top) shows mean number of endocytic structures per cell from the electron microscope images (below). Data pooled from 3 independent blocks with 5 cells each. Untreated WT MEFs (WT, i), IRSp53 null MEFs (IRSp53-/-, ii) or LG186-treated WT MEFs (LG186, iii) were incubated for 2 minutes at 37°C with 10mg/ml HRP as a fluid phase marker before processing for electron microscopy. Endocytic structures close to the plasma membrane (PM) are filled with the electron dense peroxidase precipitate. WT cells (left) show a range of endocytic structures including vesicular structures (double arrowheads) and tubular/ring-shaped putative CLIC/GEECs (large arrowheads) but the KO cells (middle) and LG186-treated (right) cells show predominant labelling of vesicular profiles. (**c**) Electron micrographs of AGS cells co-transfected GFP-IRSp53 and GBP-Apex. (**i-ii**) The reaction product is highly patched (arrowheads) on the plasma membrane (PM) along with zoomed regions on the right. (**iii**) Double arrowheads indicate specific labelling within defined microdomains of filopodia. (**d**) Plot (top) showing quantification of co-localizing IRSp53 with p21 (ARP2/3 complex subunit) using ImageJ plugin (Van Steensel’s CCF, See Methods.) when compared with its random. Representative (bottom) images of AGS cells co-expressing mEmerald-p21 subunit with mCherry-IRSp53, which were fixed and imaged with TIRFM, with zoomed inset at top left corner. Data was pooled from 2 independent experiments and the cell number is indicated below the graph. (**e**) (1a-c) Wide-field images of cells at 1x (1a), 4x (1b) along with a representative confocal slice of a cell (4x, 1c). (2a-c) Inset from the 4x cell (Supplementary Movie 5) depicting enrichment of IRSp53 (magenta, 2b) within filopodia (CD44, green, 2a) along with the merge (2c). (3-6) Insets of CD44 (green) labelled invaginations showing recruitment of IRSp53 (magenta) at various stages. Each example depicts from left to right CD44, IRSp53 and Merge respectively. Scale bar, 20µm (**a**), 1µm (**b-c**), 5µm (**d**), 20µm (**e, 1a-b**), 5µm (**e, 1c**), 1µm (**e, 2a-c & 3-6**) respectively.  *p-value* < 0.01 (*), and 0.001(**) by Mann-Whitney U Test (**d**) and 2-sample student’s T-test (**b**).


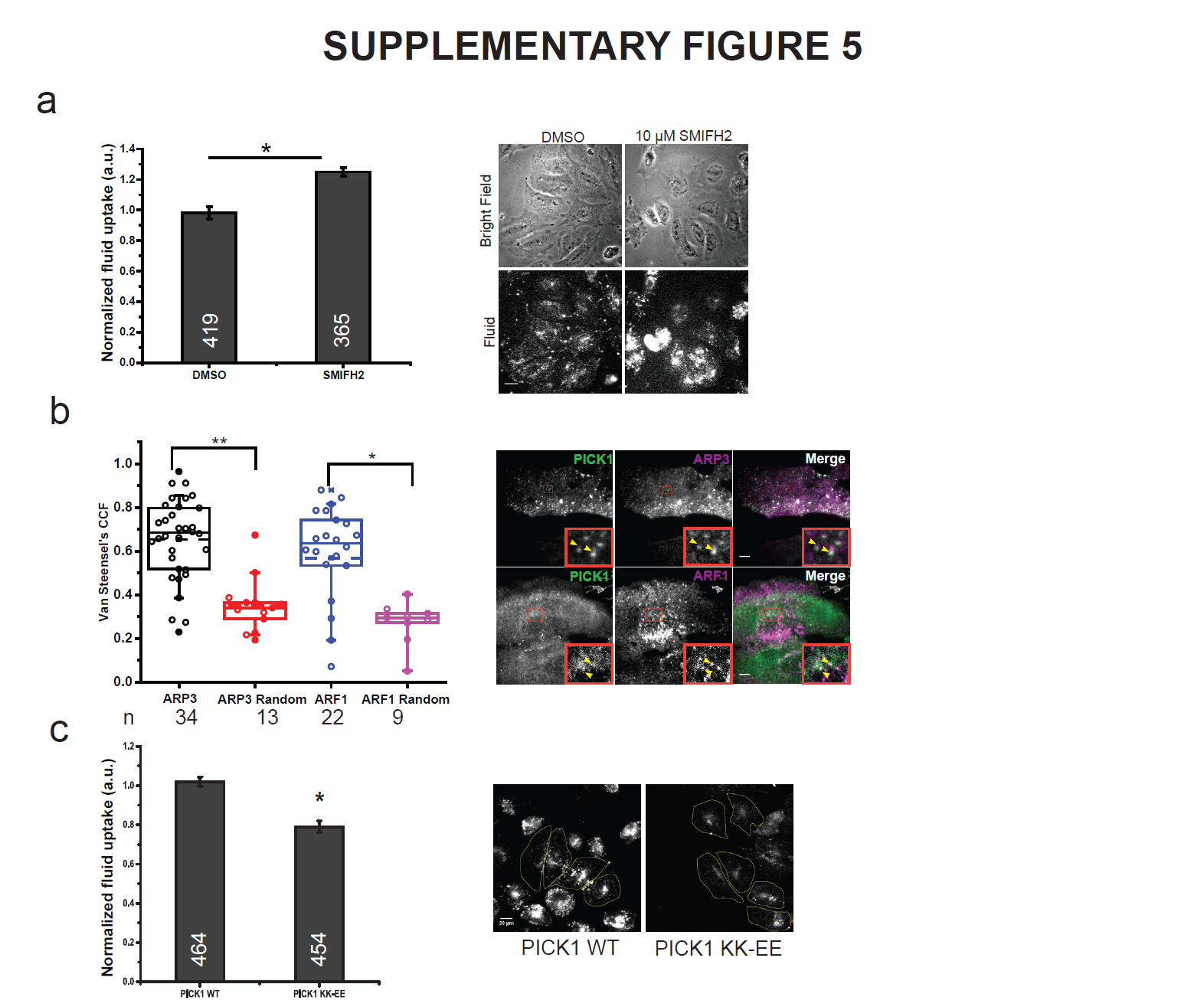


**Supplementary Figure 5:** **ARP2/3 is negatively regulated by PICK1 for CG endocytosis independent of N-WASP.**  (**a**) Histogram (left) showing quantification of 5-minute pulse fluid-phase in AGS cells treated with either DMSO or 10µM SMIFH2 along with representative images (right). Data was pooled from 2 independent experiments and the cell number is indicated in the figure. (**b**) Plot (left) showing quantification of co-localizing PICK1 with ARP3 or ARF1 using ImageJ plugin (Van Steensel’s CCF, See Methods) when compared with its random. Data was pooled from 2 independent experiments and the number the cell number is indicated below the graph. Representative (right) images of AGS cells co-expressing GFP-ARF1 with TagRFP-PICK1 or GFP-PICK1 and mCherry-ARP3 which were fixed and imaged with TIRFM, with zoomed inset at bottom right corner. (**c**) Histogram (left) showing quantification of mean 5-minute pulse fluid-phase in AGS cells overexpressing either pIRES-PICK1 WT or PICK1 KK-EE mutant. Data was pooled from 2 independent experiments and the number of cells is indicated in the figure. Error bar represents s.d. (**a**, & **c**) Scale bar, 20µm (**a** & **c**), 5µm (**b**)*. p-value* < 0.01 (*), and 0.001 (**) by Mann-Whitney U Test (**a-c**).


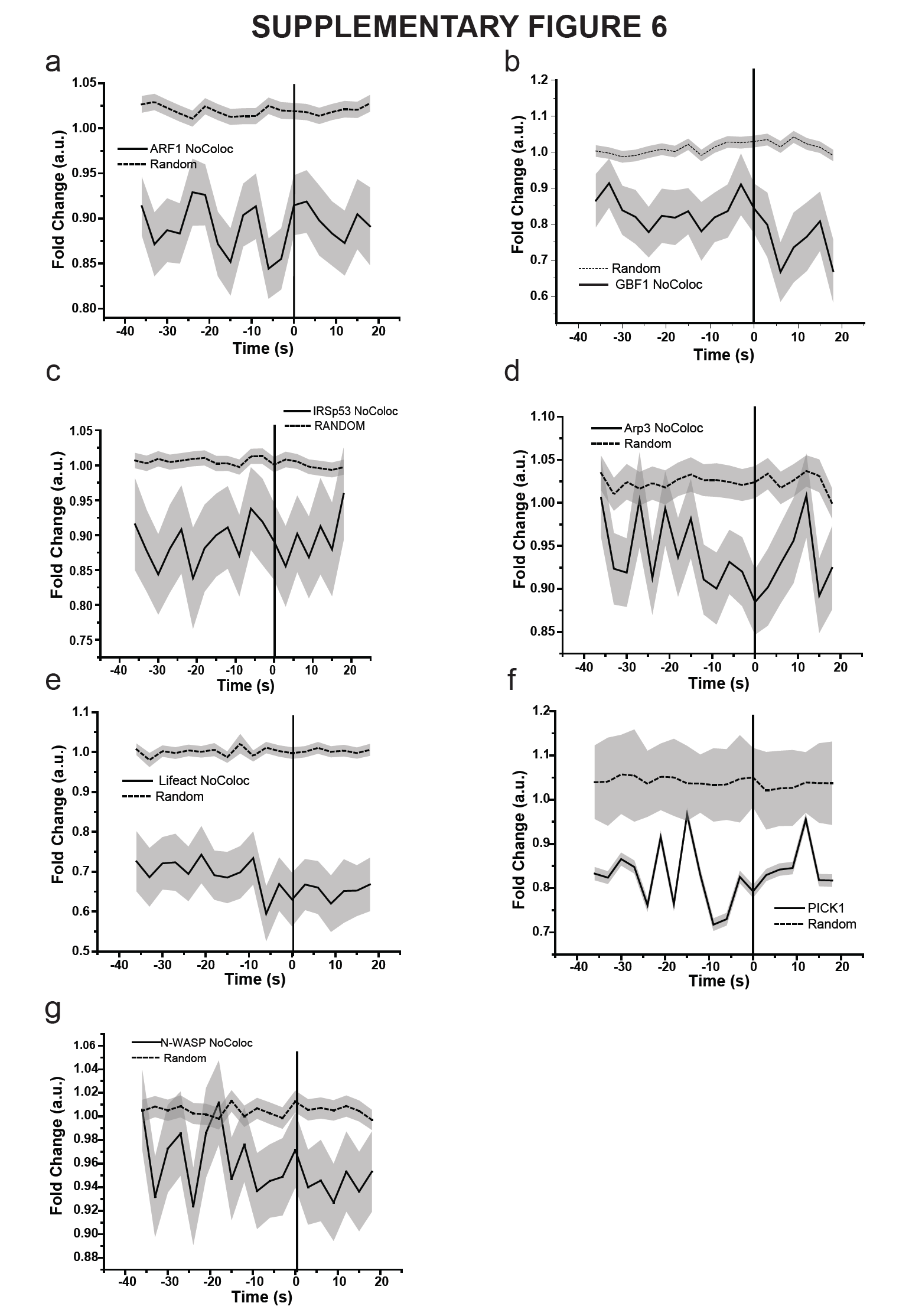
**Supplementary Figure 6: Fold change over time profiles of SecGFP-GPI spots that did not show a co-detected X-FP spot**. (**a-h**) Graphs show the average normalized fluorescence intensity versus time traces for the recruitment of mCherry-ARF1 NoColoc (**a**), mCherry-GBF1 NoColoc (**b**), mCherry-IRSp53 NoColoc (**c**), mCherry-ARP3 NoColoc (**d**), pRuby-Lifeact NoColoc (**e**), TagRFPt-PICK1 NoColoc (**f**), mCherry-NWASP NoColoc (**g**) compared to their respective random trace. The random traces were derived from randomly assigned spots of the same radius as the endocytic regions, as detailed in S.I. They represent the fraction of SecGFP-GPI that failed to show a co-detected the X-FP spots. For n see Table 1 and S.I. for further information.

**Supplementary Methods and Materials**

**Chemicals and reagents:** All reagents were purchased from Sigma unless otherwise mentioned. TMR-Dextran (10kDa) and Alexa^TM^ dyes were purchased from Molecular Probes (Eugene, OR). Transfection was carried out using FuGENE6 reagent (Promega, USA) as per the manufacturer’s instructions. LG186 was synthesized in the laboratory of Dr. Ram A. Vishwakarma (Indian Institute of Integrative Medicine (CSIR), Canal Road, Jammu 180001, India). Heat inactivated fetal bovine serum (16000044**).** Hygromycin B (10687-010) was obtained from Invitrogen. CK-666 (SML0006-5MG), SMIFH2 (S4826-5MG), FSC231 (529531-10MG, Millipore), Puromycin (P2233). PI-PLC (purified in-house, used at 50µg/ml). hTf was Iron loaded and purified in gel filtration ^1^ and was fluorescently labelled with (A 10235), Alexa-568 (A 10238) or Alexa-647 (A 20173) as per manufacturer’s instructions. Acryloyl X – SE (Life technologies: A20770). Sodium acrylate (408220, Sigma).

**Cell culture:** FR-AGS (adenogastric carcinoma cells stably transfected with FR-GPI) ^2^ were maintained in 10% FBS HF12 (HiMedia, India). S2R^+^ TfR cells ^2,3^ were grown in 10% FBS Schneider’s Drosophila Media (21720-024, Gibco^TM^). IRSp53-/- (pBABE-puro empty) and IRSp53-/-pBABE-puro-IRSp53WT mouse embryonic fibroblasts ^4^ were grown in 20% FBS DMEM (31053-028, Gibco^TM^ ) supplemented by with 1 µg/ml Puromycin. All media were supplemented with L-Glutamine Penicillin Streptomycin Solution (Sigma, G1146-100ml). CK-666 treatment was done for 90 minutes. SMIFH2 treatment was done for 60-90 minutes until lamellipodia formation was observed. FSC231 treatment was done for 60 minutes. LG186 treatment was done in serum free media for 45 minutes (MEFs) and 30 minutes (FR-AGS).

**Antibodies**: α-hTfR monoclonal antibody was purified from mouse hybridoma, OKT9 ^5^ (National Centre for Cell Science, India, used at 1:100), α-PICK1 antibody (PA1-073, Thermo Pierce, used at 1:50). α-mTfR antibody (553264, Becton Dickinson, used at 1:50) and Cy3-Mov18 (5µg/mL) ^5,6^. Fab fragments of α-GFP monoclonal antibodies generated using papain digestion subsequently fluorescently labelled with Alexa-647(used at 1:100). α-GFP antibody (ab290, abcam, 1:200). α-CD44 antibody (IM7 clone, 14-0441-82, eBiosciences, 1:150). α-IRSp53 (HPA023310, Sigma, 1:50).

**Plasmids:** SecGFP-GPI was made by site-directed mutagenesis, F64L and S65T in ecliptic-GPI ^7^ was a gift from Gero Miesenbӧck (University of Oxford). pIRES-EGFP-PICK KK-EE and pIRES-EGFP-N-WASP-CA domain were a gift from J. Hanley (University of Bristol, UK) ^8^. GFP-N-WASP-VCA and GFP-N-WASPΔVCA were a gift from Mike Way (Francis Crick Institute). ARF1-mCherry was a gift from Paul Melançon ^9^ (University of Alberta). GBF1-mCherry was a gift from Catherine Jackson (Institut Jacques Monod) ^10^. pRuby-Lifeact was a gift from Roland Wedlich-Soeldner (Max Planck Institute of Biochemistry) ^11^. IRSp53-mCherry, SecGFP-TfR ^12^, psPAX2 and pMD2.G were gifts from Dr Marcus J. Taylor (UCSF/NCBS). GFP-PICK1 and TagRFPt-PICK1 was a gift from Harvey McMahon (Medical Research Council, UK). Tag-RFPt-CDC42 was made in the laboratory by sub-cloning GFP-CDC42 into a Tag-RFPt vector. Tag-RFPt vector was purchased from Evrogen (Catalogue # FP141). GFP-IRSp53 and mutants were generated in the laboratory of Giorgio Scita (IFOM). Dyn2-pmCherryN1 (Addgene plasmid # 27689), CLC-pmCherryC1 (Addgene plasmid # 27680) and Arp3-pmCherryC1 (Addgene plasmid # 27682), mCherry-WASP-N-18 (Addgene plasmid # p55164). GFP-binding peptide conjugated to APEX2 (Addgene plasmid #67651). shRNA against hPICK1 (V3LHS_347038, RHS4531-EG9463, 5’TTCTTCAACACAATGTCCA3’) and GIPZ Non-silencing Lentiviral shRNA Control (RHS4346) were purchased from Open Biosystems.

**Generation of lentiviral stable lines:** To generate GFP-IRSp53 and its different mutant addback stable lines in IRSp53 -/- mouse embryonic fibroblasts and shRNA stable line for FR-AGS, second generation lentiviral packaging systems was used following the protocol from Trono lab ^13^. HEK293T cells was used for virus generation and PEI (polyethylenimine) was used for transfection. The ratio of DNA to PEI used for transfection is gene of interest: packaging (psPAX2): envelope (pMD2.G) in 1200ng: 1200ng: 1200ng for 3 wells of a 6-well plate. This DNA mix is added to serum free DMEM (327 µL/well) and PEI (18.8 µL/well) and the transfection mix is incubated at room temperature for 20 minutes and added to the cells. PEI is used at 1mg/mL and is heated to 70℃ for 10 minutes before transfection. Viruses were collected after 2-3 days and syringe filtered using 200 µm filter and added to 60% confluent culture. Typically after 3-4 days of infection gene expression was observed. A similar procedure was followed to generate stable shRNA lines by in FR-AGS.

**Immunostaining:** Antibodies were tested for their specificity by western blotting if it was suitable. Additionally expression was verified with published reports and was compared with the expression of the fluorescently tagged version of the gene. Cells were fixed using 4% paraformaldehyde for 20 minutes at room temperature. Cells were permeablised with 0.1% TritonX at for 10 minutes followed by blocking for 1 hour using 2mg/mL BSA in PBS. α-PICK1 was diluted in blocking buffer (1:50) and added to the cells overnight at 4°C, followed by A647-Goat-α-Rabbit for 1 hour at room temperature.

**Image Analysis**

***pH pulsing assay:*** A semi-automated analysis was developed on MATLAB (M.S.) to identify newly formed endosomes in the pH pulsing assay and trace their intensity over time in pH 7, pH 5 and RFP channels. The following steps (a-g) describe the procedure used:

1. Identification of new endocytic structures: Two consecutive pH 5 frames were segmented using MATLAB based tracking software which segments using sub-pixel localisation of particles using radial symmetry centre ^14^. The x-y coordinate of each particle in the i^th^ frame was compared with the rest of the particles in the i-1 frame and the nearest neighbour was located. If the nearest neighbour for a particle in the i^th^ frame was found in the i-1 frame within 5-pixel radius of its current co-ordinate, then that particle was rejected. If the nearest neighbour was not found within a 5-pixel radius of its current co-ordinate and it stayed on for the next frame, then that particle was considered ‘new’.
2. Intensity calculation and bleach correction: A mask of radius 3 pixels (252 nm) grown from the centroid of the new spot was used for all the calculations. For the local background, an annulus of radius 6-8 pixels (8-10 pixels for PICK1) was taken from the centroid of the new spot. The average intensity of a spot is normalised to the average intensity of the local annulus. (mean intensity of spot - 20th percentile of local background) / (mean intensity of local background - 20 percentile of local background) $\left( \frac{\sum I_{s}}{n_{s}}-20th percentile of I_{b} \right)/{\left( \frac{\sum I_{b}}{n_{b}}-20th percentile of I_{b} \right).}$Each spot was considered if its average intensity was at least 20% higher than the local background to throw out low signal to noise spots. Additionally, we demanded that in the subsequent frame the average intensity of the spot should be within 10% so rule out a spot coming into the TIRF plane, instead of it being recently pinched endocytic vesicle. A spot’s intensity will increase as it approaches the plasma membrane, while a recently pinched endocytic vesicle will not show a rise in the intensity. These newly identified structures in the TIRF plane were used as fiducial markers of the event.
3. Random spots generation: Arbitrary regions using the masks defined in step (b) within the cell boundary was chosen. The intensity of such spots is henceforth called “Random” was calculated as discussed in Step (b) and was used to compare the behaviour of X-FP at the new SecGFP-GPI spots vs. random locations in the cell.
4. False positive removal: In addition to the criteria mentioned in Step (b), separately, montages of each event depicting frames from -18s to +18s were generated and used to manually verify the appearance of a new event. In addition to it, a spot can be mistakenly identified as new due to the sudden lateral movement into the frame or growth of a sub-threshold spot, appearing in the TIRF plane from the inside of the cell. These events are screened out by manually verifying the absence of such occurrences in the frames prior to 0s in the montages.
5. Manual classification of the X-FP spots: In addition to removing the false positives, the montages generated at the Step (d) underwent manual check to classify the new SecGFP-GPI spots into two groups based on whether X-FP co-detection was observed or not. One population exhibited co-localisation of X-FP with SecGFP-GPI during the time window of -18 to +18s for at least 1 frame, while the second failed to register any co-localization. We called them X-FP Coloc (Co-detection of X-FP and SecGFP-GPI) and X-FP NoColc (the remainder). The X-FP NoColoc profile was comparable to Random (Supplementary Fig.2b and 6) indicative of lack of recruitment in that fraction. This was supported by X-FP All and X-FP Coloc having similar profiles for most CG pathway molecules (Compare Supplementary Fig.2a and Fig. 1d). Although the fraction of X-FP NoColc was variable, we observed that for molecules that were involved in CG endocytosis, it was typically between 20-40%. On the other hand, for the non-CG pathway molecules like Clathrin, Dynamin and N-WASP it was around 60-70%. Thus, the endocytic sites detected by our assay consisted of two populations wherein one fraction exhibited an accumulation of X-FP while the second fraction failed to show a discernable accumulation. As the removal of the events that did not coincide with the presence of X-FP did not alter their recruitment profile, they were discarded from further analysis.

Despite removing, the false positive via both automatic and manual methods there exists a fraction of SecGFP-GPI endocytic sites that fails to associate with X-FP. The reasons for not detecting X-FP at every endocytic event is both a function of both the signal and noise in the data, and it reflects a genuine lack of recruitment at some endocytic events.

The number of SecGFP-GPI spots obtained for every molecule, the fraction of X-FP that were associated with the SecGFP-GPI spots, the number of random spots along with the number of cells and experiments is detailed in **Table 1**.

1. Plotting: Intensity, corrected for bleaching and normalised, was plotted against time for each event and then averaged across all spots. The average data was compared to average intensity obtained from multiple randomly picked regions from inside the mask of the cell. The random data points ranged from 1800 to 5000. Y-axis represents fold change over the local background. All the pH pulsing trace used circular mask of radius 3 pixels (250 nm) whose mean intensity was normalized to the local background mask donut of size 6-8 pixels (470-672 nm) except for IRSp53 (additional masks of circular radius 2 pixels, (168 nm), donut 3-5 pixels (250-420 nm normalized to background donut 6-8 pixels (470-672 nm)) and PICK1 which was normalized to background donut 8-10 pixels (672-840 nm).
2. Correlation analysis: The average intensity trace over time between two molecules was compared by using MATLAB function called ‘corrcoef’ that calculates Pearson correlation coefficient wherein the *p*-value is calculated by *t*-statistic.

**Microscope:** For population-based endocytic assays quantification of uptake of endocytic tracers, images were obtained using either low magnification objective. For pH pulsing assay the imaging was performed in a TIRF setup with custom designed chamber to maintain constant temperature. The imaging was done at 30°C. For residence time imaging the imaging was done on a TIRF setup at 37°C. Confocal imaging was done with spinning disk setup.

A detailed list of microscopes used for this manuscript is below.

1. Nikon TE300 - Objectives used in this setup were 20x 0.75 NA, 40x 0.65 NA. The images collected using an EMCCD camera Ixon (Andor Technologies) using µManager ^15^. This microscope was utilized for population level endocytic assay imaging.
2. Nikon TE 2000 equipped with TIRF- Objective used in this setup were 20x 0.75 NA, 40x 0.65 NA and 100x 1.49 NA oil. The camera in this setup is CCD cascade camera (Photometrics Inc., USA). Images were acquired using Metamorph^TM^ and µManager. This microscope was utilized for pH pulsing assays, residence time, TIRF based co-localization and population level endocytic assay imaging.
3. Nikon Eclipse Ti equipped with TIRF setup fed by Agilent laser combiner MCL400 (Agilent technologies). Laser lines used were 488 and 561 along with epi-fluorescence lamp. Objectives used in this setup were 20x 0.75 NA, 40x NA and 100x 1.49 NA objective with EMCCD camera (Photometrics Inc., USA). Image acquisition was done with µManager. This microscope was utilized for residence time, TIRF based co-localization and population level endocytic assay imaging.
4. Nikon Eclipse Ti equipped with confocal spinning disk unit (Yokogawa CSU-22 scan head). Images were collected with 100x 1.4 NA oil Nikon objective with an EMCCD camera (Andor ixon+897). Images were acquired using Andor iQ2 with Python scripting. This microscope was utilized for confocal imaging.
5. Perkin Elmer equipped with confocal spinning disk unit (Yokogawa CSU-X1 scan head). Images were collected with 100x 1.4 NA oil Olympus objective with Hamamatsu IMAGE EM X2 EMCCD camera. Images were acquired using Volocity software. This microscope was utilized for confocal imaging.

**Supplementary References**

1. Mayor, S., Presley, J. F. & Maxfield, F. R. Sorting of Membrane-Components From Endosomes and Subsequent Recycling To the Cell-Surface Occurs By a Bulk Flow Process. *J. Cell Biol.* **121,** 1257–1269 (1993).

2. Gupta, G. D. *et al.* Population distribution analyses reveal a hierarchy of molecular players underlying parallel endocytic pathways. *PLoS One* **9,** (2014).

3. Gupta, G. D. *et al.* Analysis of endocytic pathways in Drosophila cells reveals a conserved role for GBF1 in internalization via GEECs. *PLoS One* **4,** (2009).

4. Disanza, A. *et al.* CDC42 switches IRSp53 from inhibition of actin growth to elongation by clustering of VASP. 2735–2750 (2013). doi:10.1038/emboj.2013.208

5. Kumari, S. & Mayor, S. ARF1 is directly involved in dynamin-independent endocytosis. *Nat. Cell Biol.* **10,** 30–41 (2008).

6. Coney, L. R. *et al.* Cloning of a tumor-associated antigen: MOv18 and MOv19 antibodies recognize a folate-binding protein. *Cancer Res.* **51,** 6125–6132 (1991).

7. Miesenböck, G., De Angelis, D. A. & Rothman, J. E. Visualizing secretion and synaptic transmission with pH-sensitive green fluorescent proteins. *Nature* **394,** 192–5 (1998).

8. Rocca, D. L., Martin, S., Jenkins, E. L. & Hanley, J. G. Inhibition of Arp2/3-mediated actin polymerization by PICK1 regulates neuronal morphology and AMPA receptor endocytosis. *Nat. Cell Biol.* **10,** 259–71 (2008).

9. Chun, J., Shapovalova, Z., Dejgaard, S. Y., Presley, J. F. & Melançon, P. Characterization of class I and II ADP-ribosylation factors (Arfs) in live cells: GDP-bound class II Arfs associate with the ER-Golgi intermediate compartment independently of GBF1. *Mol. Biol. Cell* **19,** 3488–500 (2008).

10. Bouvet, S., Golinelli-Cohen, M.-P., Contremoulins, V. & Jackson, C. L. Targeting of the Arf-GEF GBF1 to lipid droplets and Golgi membranes. *J. Cell Sci.* **126,** 4794–805 (2013).

11. Riedl, J. *et al.* Lifeact: a versatile marker to visualize F-actin. *Nat. Methods* **5,** 605–7 (2008).

12. Taylor, M. J., Perrais, D. & Merrifield, C. J. A high precision survey of the molecular dynamics of mammalian clathrin-mediated endocytosis. *PLoS Biol.* **9,** e1000604 (2011).

13. Barde, I., Salmon, P. & Trono, D. Production and titration of lentiviral vectors. *Curr. Protoc. Neurosci.* 1–23 (2010). doi:10.1002/0471142301.ns0100s37

14. Parthasarathy, R. Rapid, accurate particle tracking by calculation of radial symmetry centers. *Nat. Methods* **9,** 724–726 (2012).

15. Stuurman, N., Edelstein, A. D., Amodaj, N., Hoover, K. H. & Ronald, D. Computer Control of Microscopes using μ Manager. 1–22 (2011). doi:10.1002/0471142727.mb1420s92.Computer
