# Supplementary material for: Small GTPases and BAR domain proteins regulate branched actin polymerisation for clathrin and dynamin-independent endocytosis
Source: Nat Commun. 2018 May 9;9:1835. doi: 10.1038/s41467-018-03955-w (PMC5943408; doi:10.1038/s41467-018-03955-w)
Supplement: Supplementary file 3 — Description of Additional Supplementary Files [file 41467_2018_3955_MOESM3_ESM.pdf]

Description of Additional Supplementary Files

File Name: Supplementary Movie 1
Description: Representative pH pulsing movie. AGS cells co-transfected with SecGFP-GPI and mCherry-ARF1 were imaged as described (See, S.I., pH pulsing assay). The yellow circles depict when a new event is detected. Three, movies are stitched together, where SecGFP-GPI/pH 7 (left), SecGFP-GPI/ pH 5 (middle) and mCherry-ARF1/both pH 5 & pH 7 (right) may be visualized.

File Name: Supplementary Movie 2
Description: Representative event of nascent SecGFP-GPI endosome detection (zoomed from Supplementary Movie 1). AGS cells co-transfected with SecGFP-GPI and mCherry-ARF1 were imaged as described (See, S.I., pH pulsing assay). The yellow circles depict when a new event detection. 3 movies are stitched together, SecGFP-GPI/pH 7 (left), SecGFP-GPI/ pH 5 (middle) and mCherry-ARF1/both pH 5 & pH 7 (right).

File Name: Supplementary Movie 3
Description: 3D-Tomogram of APEX based detection of GFP-IRSp53 on CLICs (Representative movie 1). AGS cells co-transfected with APEX-GBP and GFPIRSp53 were processed and imaged as described (See, S.I. Electron Microscopy). The DAB reaction was performed and the cells were processed for electron tomography (See S.I.). The first half of the movie depicts sections of the original tomogram followed by density-based thresholded sections. The whole of PM of the tomographic volume was rendered and enlarged tubular regions of interest show GFP-IRSp53 recruitment.

File Name: Supplementary Movie 4
Description: 3D-Tomogram of APEX based detection of GFP-IRSp53 on CLICs (Representative movie 1). AGS cells co-transfected with APEX-GBP and GFPIRSp53 were processed and imaged as described (See, S.I. Electron Microscopy). The DAB reaction was performed and the cells were processed for electron tomography (See S.I.). The first half of the movie depicts sections of the original tomogram followed by density-based thresholded sections. The whole of PM of the tomographic volume was rendered and enlarged tubular regions of interest show GFP-IRSp53 recruitment.

File Name: Supplementary Movie 5
Description: Representative 3-D confocal (0.3µm step) stack of 4x expanded cell depicting CD44 (green) and IRSp53 (magenta). The regions showing invaginations and filopodia are marked. Scale bar represents 5µm.
